# Physiological and subjective evaluation of PPE using a sweating thermal manikin

**DOI:** 10.1186/2046-7648-4-S1-A27

**Published:** 2015-09-14

**Authors:** Aitor Coca, Travis Dileo, Jung-Hyun Kim, Raymond Roberge, Ronald Shaffer

**Affiliations:** 1 National Personal Protective Technology Laboratory of the National Institute for Occupational Safety and Health, Pittsburgh, PA, USA

## Introduction

Experience with personal protective equipment (PPE) ensembles used by healthcare workers (HCWs) during the Ebola outbreak in the hot, humid conditions of West Africa has prompted significant concerns with heat stress and the inability to work in the PPE for extended work periods.

## Methods

A sweating thermal manikin was used to ascertain the time to achievement of a critical core temperature of 39 °C while wearing four different PPE ensembles (consisting of various types of coveralls, or surgical gowns in addition to other protective clothing layers such as aprons, hoods, googles and gloves) similar to those suggested by the World Health Organization (WHO) and Mèdecines Sans Frontiéres/Doctors Without Borders (MSF), at two different ambient conditions (32 °C / 92 % rh and 26 °C / 80 % rh) compared with control ensembles (medical scrubs and rubber boots).

## Results

Sweating thermal manikin data indicated that the PPE ensembles 3 and 4 with moderate-to-high degrees of impermeability increase T_co _to a critical level of 39 °C more rapidly than the control, and ensembles 1 and 2. Encapsulation of the head and neck region resulted in higher model predicted subjective impressions of heat sensation (Table 1).

**Table 1:** Mean (SD) time to reach core temperature (T_co_) of 39 °C in the five ensembles for Condition A (32 **°**C and 92 % rh) and skin temperature (T_sk_), comfort, and heat sensation at that point; T_co_, T_sk_, comfort and heat sensation for Condition B (26 **°**C and 80 % rh) at 80 min of testing.

T_co _(°C)	PPE	Condition	TIME (min)	T_sk _(°C)	Heat Sensation	Comfort
**39**	Control	**A**	+80^b,c,d,e^	36.8(0.2)^c,d,e^	3.2(0. 1)^d,e^	-3.1(0.1)^d,e^

	Ensemble 1	**A**	+80^a,d,e^	37.3(0.3)^d,e^	3.6(0.2)	-3.2(0.1)

	Ensemble 2	**A**	78(7)^a,d,e^	37.7(0.2)^a^	3.5(0.2)	-3.2(0.1)

	Ensemble 3	**A**	65(3)^a,b,c^	38.3(0.2)^a,b^	3.7(0. 1)^a^	-3.4(0.1)^a^

	Ensemble 4	**A**	62(5)^a,b,c^	38.4(0.8)^a,b^	3.8(0. 1)^a^	-3.4(0.1)^a^

**TIME (min)**			**T_co _(°C) at 80 min**			

**80 min**	Control	**B**	37.9(0.1)	35.4(0.2)	2.6(0.5)	-1.8(0.4)

	Ensemble 1	**B**	38.05(0.1)^c,d,e^	35.8(0.6)^e^	2.4(0.5)^e^	-2.3(0.3)^e^

	Ensemble 2	**B**	38.33(0.1)^b,d,e^	36.4(0.4)^e^	2.5(0.6)	-2.6(0.4)^e^

	Ensemble 3	**B**	38.7(0.1)^b,c^	36.9(0.2)	2.5(0.4)	-3(0.2)

	Ensemble 4	**B**	38.9(0.2)^b,c^	37.6(0.4)^b,c^	3.2(0.6)^b^	-3.2(0.2)^b,c^

(n = 3)

Control PPE for Condition B was not included in the statistical analysis because only two replicates were collected. Superscripts indicate pairs of values that differ significantly (P < 0.05) where a=Control, b=Ensemble 1, c=Ensemble 2, d=Ensemble 3, and e=Ensemble 4.

## Discussion

Ensemble configurations similar to the ensemble 4 PPE studied here are currently in use by MSF healthcare personnel in Ebola-affected countries of West Africa. The present study indicates that use of this ensemble results in significant heat stress after one hour of use in a "near worst case" ambient environment scenario (32 °C, 92 % RH) at a typical HCW work rate (3 METs).

## Conclusion

Implementation of appropriate work/rest ratios is recommended for HCWs in West Africa when wearing PPE ensembles similar to those studied here, as well as investigating possible cooling strategies and other precautions that would alleviate the heat stress faced by HCW. These measures will help achieve thermal relief during the recovery periods and allowing possibly longer, but safer, work periods. The subjective impact of head/neck encapsulation on heat perception requires further investigation and could potentially be ameliorated by the use of alternative equipment (e.g., powered air-purifying respirators with shrouds, and/or more breathable materials for body and head protection).

## Disclaimer

The findings and conclusions in this paper are those of the authors and do not necessarily represent the views of CDC. Mention of product names does not imply endorsement. The authors identify no conflicts of interest in the conduct of this study.

